# Factors influencing water immersion during labour: qualitative case studies of six maternity units in the United Kingdom

**DOI:** 10.1186/s12884-020-03416-7

**Published:** 2020-11-23

**Authors:** Sarah Milosevic, Susan Channon, Jacqueline Hughes, Billie Hunter, Mary Nolan, Rebecca Milton, Julia Sanders

**Affiliations:** 1 Centre for Trials Research, College of Biomedical and Life Sciences, Cardiff University, Neuadd Meirionnydd, Heath Park, Cardiff, Wales CF14 4YS; 2 School of Healthcare Sciences, College of Biomedical and Life Sciences, Cardiff University, Eastgate House, 35-43 Newport Road, Cardiff, Wales CF24 0AB; 3 College of Health, Life and Environmental Sciences, University of Worcester, Henwick Grove, Worcester, England WR2 6AJ; 4 School of Healthcare Sciences, College of Biomedical and Life Sciences, Cardiff University, Heath Park Campus, Cardiff, Wales CF14 4XN

**Keywords:** Water birth, Midwifery, Obstetrics, Qualitative research, Case studies

## Abstract

**Background:**

Water immersion during labour can provide benefits including reduced need for regional analgesia and a shorter labour. However, in the United Kingdom a minority of women use a pool for labour or birth, with pool use particularly uncommon in obstetric-led settings. Maternity unit culture has been identified as an important influence on pool use, but this and other possible factors have not been explored in-depth. Therefore, the aim of this study was to identify factors influencing pool use through qualitative case studies of three obstetric units and three midwifery units in the UK.

**Methods:**

Case study units with a range of waterbirth rates and representing geographically diverse locations were selected. Data collection methods comprised semi-structured interviews, collation of service documentation and public-facing information, and observations of the unit environment. There were 111 interview participants, purposively sampled to include midwives, postnatal women, obstetricians, neonatologists, midwifery support workers and doulas. A framework approach was used to analyse all case study data.

**Results:**

Obstetric unit culture was a key factor restricting pool use. We found substantial differences between obstetric and midwifery units in terms of equipment and resources, staff attitudes and confidence, senior staff support and women’s awareness of water immersion. Generic factors influencing use of pools across all units included limited access to waterbirth training, sociodemographic differences in desire for pool use and issues using waterproof fetal monitoring equipment.

**Conclusions:**

Case study findings provide new insights into the influence of maternity unit culture on waterbirth rates. Access to pool use could be improved through midwives based in obstetric units having more experience of waterbirth, providing obstetricians and neonatologists with information on the practicalities of pool use and improving accessibility of antenatal information. In terms of resources, recommendations include increasing pool provision, ensuring birth room allocation maximises the use of unit resources, and providing pool room environments that are acceptable to midwives.

**Supplementary Information:**

The online version contains supplementary material available at 10.1186/s12884-020-03416-7.

## Background

Guidelines for intrapartum care in the United Kingdom (UK) state that clinicians should offer women at low risk of complications the opportunity to labour in water [1]. Pool use is associated with reduced need for regional analgesia [2–4] and significantly shorter duration of labour [4–6]. Women using water immersion tend to report a more positive experience of labour and birth [7]; less pain and a greater sense of control [8]; and increased comfort and relaxation [9]. Research suggests using a pool during labour is safe for mothers and babies, with no evidence of increased adverse outcomes [10–13]. However, due to relatively small sample sizes and the infrequency of adverse clinical events, there are no conclusive data on the safety of birth in water [14]. Pool use has gradually increased in the UK in recent years [15], but currently less than a fifth of women in England report using water during labour (19%) or giving birth in water (11%) [16].

Research exploring the use of water immersion in the UK is limited, but multiple barriers to pool use have been identified internationally. Negative attitudes from medical staff are widely reported, primarily due to a lack of experience and concerns about safety [17–20]. Insufficient knowledge and education is a key issue: for example, pool use for labour and birth is commonly not included in Australian midwifery training [21]; in a Swedish survey of healthcare professionals, 26% of midwives and 38% of obstetricians/gynaecologists reported no knowledge of how to assist a waterbirth [20]. Research examining Australian water immersion policies and guidelines has found them to be restrictive and unsupportive [22], often mandating that midwives meet accreditation requirements (such as additional training) before being allowed to facilitate pool use [23]. Further barriers to water immersion include inadequate staffing levels [18, 19], poor information provision antenatally [24] and stigma from women’s family and friends (e.g. comments that waterbirth is reckless and unsafe) [25].

In the UK, depending on local facilities, women can give birth in an obstetric unit (86% of births in England), an alongside midwifery unit (12%), a freestanding midwifery unit (2%) or at home (2%) [26]. Women with medical or obstetric risk factors are usually advised to plan birth in an obstetric unit. Pool use, even among women without risk factors, is significantly lower amongst women who plan to give birth in an obstetric unit (9%) compared with those planning to give birth in a freestanding (46%) or alongside (30%) midwifery unit [27]. Research indicates that as found in other countries, staff attitudes and behaviour are also key influences on pool use in the UK. An action research study of an obstetric unit in England [28, 29] revealed that midwives’ negative attitudes were perceived as a key barrier to pool use. Senior staff and unit practices were unsupportive of water immersion, so it was not routinely offered to women. A further study [30] highlighted that while water immersion is often restricted in obstetric units, women giving birth in midwifery units tend to be supported and encouraged to use a pool.

No published studies have qualitatively explored the differences between UK maternity units in relation to pool use. The international research suggests unit culture - which includes staff attitudes, beliefs and behaviour, as well as guidelines relating to water immersion practices - is critical. Therefore, it was important that the design of this study enabled these aspects to be examined in depth. We aimed to identify factors influencing water immersion during labour through case studies of three obstetric units and three midwifery units in the UK.

## Methods

### Study design

This research forms part of the larger POOL study, a cohort study aiming to establish whether for low risk women who use a pool during labour, waterbirth is as safe for mothers and infants as leaving a pool prior to birth. The qualitative component of the POOL study has two stages. Stage one [30] gathered experiences of birth pool provision and use from the perspectives of women, midwives and medical staff via online discussion groups and telephone interviews. Findings have informed data collection for stage two, a more in-depth study of birth pool use, described in this paper.

A case study approach allows for the collection of multiple sources of data and is particularly suited to exploratory research seeking to explain a contemporary issue in depth [31]. Therefore, this method was considered appropriate to examine pool use in a real-world context and from a range of perspectives. Methods of data collection included semi-structured interviews, the collation of service documents and public-facing information, and observations of the unit environment. Ethical approval for the study was obtained from Wales Research Ethics Committee 3.

### Sampling and recruitment

Three case study sites were selected from NHS (National Health Service) organisations (trusts or health boards) participating in the POOL study that had a midwifery unit (MU) and an obstetric unit (OU) and at least one birth pool in each (see Table 1). This approach enabled a holistic exploration of the culture of maternity services at each organisation, as the relationship and differences between the midwifery unit and obstetric unit could be examined.

**Table 1:** Case study site characteristics

	Site A		Site B		Site C	
	MU	OU	MU	OU	MU	OU
Births per year (circa)	400	5000	1000	4000	600	4000
Birth rooms	3	12	4	12	5	12
Pools	2	2	4	1	3	1
Waterbirth rate (circa)	61%	5%	30%	1%	28%	0.5%

In the UK, midwifery units offer midwife-led intrapartum care to women with uncomplicated pregnancies. Alongside midwifery units are located on the same site as an obstetric unit, while freestanding midwifery units are located on a separate site. Women assessed as being at high risk of complications are advised to give birth in an obstetric unit in hospital, where care is provided by midwives and obstetricians, and services such as neonatal and anaesthetic care are available on site [32].

Purposive sampling enabled selection of sites situated in geographically diverse locations, with differing waterbirth rates. Waterbirth rates for individual obstetric and midwifery units were not routinely collected by study sites, so we requested each site to provide us with an ad hoc data extraction. Once potential sites were identified, the Principal Investigator for the wider POOL study at each site was provided with information about the case study research and invited to take part. One proposed case study site declined to participate and was replaced by a site with a similar waterbirth rate.

Site A comprised an obstetric unit with a combination of obstetric-led and midwifery-led birth rooms, and a freestanding midwifery unit located over 10 miles from the obstetric unit. Sites B and C each comprised an obstetric unit and an alongside midwifery unit located within the same hospital building. Sites A and B serve communities less deprived than the national average, while Site C serves communities in the 10% of most deprived areas nationally [33, 34]. The ethnic minority population is 11% in areas served by Site A, 16% in areas served by Site B, and 23% in areas served by Site C (compared to 14% in England and Wales as a whole) [35].

Within each case study site, sampling for interview participants was purposive, to capture a range of views from those with relevant experience. Interviews were conducted with healthcare professionals who worked in each unit (including unit midwives, community midwives, student midwives, obstetricians, neonatologists/paediatricians and midwifery support workers), women who had given birth at the unit in the previous 6 months, and doulas with experience of supporting women who had given birth at the unit.

Study researchers visited case study units and postnatal clinics and approached staff members and postnatal women directly to invite them to take part, emphasising that participation was voluntary. Doulas local to each unit were identified through internet searches and invited to take part by telephone or email. The study was also advertised via local networks, with potential participants requested to contact the research team to receive further information.

### Data collection

Separate interview topic guides were developed for each participant group, informed by the findings of stage one of the qualitative study. Interview topic guides are provided as supplementary online material. Interviews comprised open questions exploring topics such as attitudes towards pool use, unit resources, criteria for pool use and information provided to women. Supplementary follow-up questions enabled more in-depth exploration of key themes that emerged as data collection in each site progressed. This iterative and responsive approach enabled data saturation to be achieved as far as possible.

Interviews were conducted one-to-one or in small groups of up to three participants, from July 2019 to March 2020, and were undertaken by experienced qualitative health researchers (SM, JH or SC). Written informed consent was obtained for face-to-face interviews; consent was obtained verbally and audio-recorded for telephone interviews. With consent, all interviews were audio-recorded and transcribed verbatim.

Pool use guidelines, website pages and antenatal information leaflets were collated from case study sites. Researchers also documented the physical environment of the unit, using field notes and photographs.

### Data analysis

Verbatim interview transcripts, together with all documents and data collected from each case study unit, were uploaded to NVivo 12 for framework analysis. An analytic framework was initially developed deductively based on themes identified in stage one of the qualitative research, which was then used for the systematic coding of all data. This framework was updated throughout the analysis period to take account of previously unidentified themes emerging from the data. All interviews were coded by SM, with a sample of 10% independently coded by JH, to enhance reliability and validity. Once coding was complete, data from each case study unit relating to the key themes was tabulated by SM to facilitate comparison. Key influences on water immersion were identified and agreed through discussion with SC.

## Results

There were 111 interview participants across the six case study units (see Table 2). Interviews took 3–64 min (mean 21.0). Most were face-to-face, with two conducted by telephone.

**Table 2:** Interview participants

	Site A		Site B		Site C	
	OU	MU	OU	MU	OU	MU
Band 5–6 midwives	6	3	4	4	5	3
Band 7–8 (senior) midwives	6	4	1	2	2	1
Student midwives	1	4	2	2	2	1
Maternity care assistants	2	3	1	1	–	–
Postnatal women	1	1	4	6	5	4
Community midwives		6	–	4	–	6
Obstetricians	4		–		4	
Neonatologists/Paediatricians	2		–		2	
Doulas		–		2		–

In presenting unit-level factors influencing water immersion, we focus on the differences between case study units: firstly differences across case study sites, and secondly differences between obstetric and midwifery units. However, before describing the differences, it is important to highlight areas of similarity across sites, as these are systemic factors that will influence the overall rate of waterbirths in the UK. All quotes in this section are labelled with a participant identification number.

### Similarities across case study units

#### Limited access to waterbirth training

Most midwives (across all case study sites) had received no formal waterbirth training; it was seen as a ‘luxury’ that could not be fitted in alongside mandatory courses. Some had attended waterbirth study days and found these beneficial, but had funded these themselves and completed them in their own time. Student midwives had mixed exposure to waterbirth: it was not always included in theoretical-based training, placements were more often on obstetric units than midwifery units, and it was possible to complete midwifery training having never seen a waterbirth. Obstetricians also had little or no experience related to waterbirth and described their knowledge as very limited. Midwives suggested they would feel more supported in their practice if doctors had greater awareness of the benefits of pool use.

*‘I’ve never seen [a pool] used … actually … I don’t really even know how it would work. It’s not something we get taught about.. like I wouldn’t know what to do if someone had a problem in the pool. I’d just pull the plug out and drain them, or do you get the patient out and then pull the plug? I don’t know’* (Site C, Obstetric unit, Obstetrician, 416)

#### Sociodemographic differences in pool use

Midwives across all case study sites reported that white, middle-class women who are well-informed about their options are more likely to use a pool. Midwives felt this group were more likely to have written a birth plan, attended paid-for antenatal or hypnobirthing classes, and conducted their own research.

*‘I don’t want to say married, you know, nuclear family type professionals, but I do think you see an increased trend [for pool use amongst this group]. The ones who would have gone to the breastfeeding classes, the ones who paid to go to [antenatal] classes’* (Site B, Obstetric unit, Student Midwife, 105)

Women from ethnic minority (particularly Asian) communities were perceived by midwives as being less likely to use a pool. Midwives suggested this may be due to unfamiliarity with waterbirth, reluctance to remove clothing, or the views of relatives attending the birth.

*‘We would like our Asian community to try [the pool] more, but I think it’s just their culture a lot of the time [that stops them]. Probably getting undressed, to be honest … and they tend to have a lot of people with them, you know like sister-in-laws, mother-in-laws’* (Site C, Midwifery unit, Band 6 Midwife, 426)

Midwives suggested that ensuring all women were fully informed and familiar with their options antenatally could help reduce sociodemographic differences in pool use.

#### Issues using waterproof fetal monitoring equipment

Each case study obstetric unit had waterproof equipment for continuous fetal monitoring, but it was not often used and not all midwives or consultants were aware it was available. The equipment was not always charged, was sometimes not working or not synchronised to centralised fetal monitoring systems and was described as awkward to use in the pool. Midwives’ reluctance to facilitate continuous fetal monitoring in water meant women who required this were usually prevented from accessing water immersion.

*‘If you’re high risk and you need to be continuously monitored, we generally say not the pool. But then we do also have the facility for telemetry so we can use it whilst in the pool. Which I’ve actually never seen in my training or since I’ve qualified but I know people do use it’* (Site A, Obstetric unit, Band 5–6 Midwife, 204)

### Differences between case study sites

We identified key differences between the three case study sites in relation to criteria for pool use, equipment and resources, senior staff support, and women’s awareness of and attitudes towards water immersion. These are outlined here in terms of barriers and facilitators to pool use (see Table 3). Most themes were derived from interview data. Themes relating to criteria for pool use were additionally derived from examination of unit policies and guidelines; themes relating to use of equipment and resources were additionally derived from observations of the unit environment.

**Table 3:** Differences between case study sites

Categories and themes	Barriers	Facilitators
*Criteria for pool use*		
Criteria for using the pool	Women must meet specific criteria to use a pool. Lots of exclusion criteria. (*Site C*)	Few contraindications to pool use mentioned in guidelines; risk factors assessed on an individual basis (*Site A*) Guidelines specifically mention women with risk factors who can use a pool (*Site B*)
Staff training requirements	Midwives required to be trained to support women in water for labour/ birth; otherwise they have to approach their manager before undertaking this (*Site C*)	No specific training required (other than emergency evacuation training); midwives required to have the necessary competences and skills (*Sites A & B*)
Criteria for entering the pool	Women should be in established labour (4–5 cm) (*Sites B & C*)	No fixed cervical dilation required; pool can be used for prolonged latent phase (*Site A*)
*Use of equipment and resources*		
Allocation of pool rooms	Women usually automatically allocated a non-pool room (*Site B, Obstetric unit*) Pool room used for non-water births so is rarely empty (*Site C, Obstetric unit*) Women not using the pool would not be moved out of the pool room during labour (*Site C, Obstetric unit*)	Low risk women asked at triage if they would like to use a pool (*Site A, Obstetric unit*) Some midwives will ask women to switch rooms to free up the pool room (*Site A, Obstetric unit*)
Filling pool	Pool is usually filled after women arrive (*Sites B & C, Midwifery unit*)	Pool is automatically run when women are on their way (*Site A, Midwifery unit*)
Pool room environment	Pool rooms less popular than other rooms (*Sites B & C, Obstetric unit*)	Considered ‘nicer’ than general birth rooms; one pool room located near midwives’ station (*Site A, Obstetric unit*)
Emergency procedures	Emergency evacuation not well-practised; some midwives not confident in emergency procedures (*Site C, Obstetric unit*)	Midwives confident in ability to cope with emergencies in the pool (*Sites A & B, Obstetric unit*)
Home birth pool use	Cost can be a barrier to pool hire (*Sites A & B*) Women have to source their own pool (*Sites A & B*)	Pools, liners and water pumps provided for women wanting to use a pool at home (*Site C*)
*Support for natural birth*		
Support for natural birth	Obstetric consultants sometimes block women from accessing the midwifery unit (*Site C*)	Obstetric consultants are supportive of the midwifery unit and facilitate women going there (*Sites A & B*) Support for natural birth at all levels of management (*Site A*)
*Women’s awareness of and attitudes towards pool use*		
Tours of the unit	No tours of the midwifery or obstetric unit; virtual tour available online (*Site B*)	Women are invited to have a tour of the midwifery unit (*Sites A & C*)
Antenatal classes	Antenatal classes are over-subscribed, so not all women have the chance to attend (*Sites A & B*)	Antenatal classes are available for all women and pool use is discussed at length (*Site C*)
Sociodemographic differences	Large Asian population who are perceived as less likely to want to use the pool (*Site C*)	Women in the area are well-informed and aware of their options (*Sites A & B*)

#### Criteria for pool use

Despite differences between case study sites (see Table 3), midwives at all units felt guidelines were appropriate and broadly supportive of pool use. However, they were applied inconsistently, with adherence depending on the knowledge and attitudes of individual clinicians; for example, midwives had mixed views as to whether women should enter the pool in early labour.

*I think if people followed the guidelines on who can use the pool a bit more, then they would be helpful. Like … up on the high-risk unit now, if they thought about who could actually labour in the pool … But … it’s not really offered, even to the women who can use it’* (Site B, Midwifery unit, Student Midwife, 109)

*‘It varies from consultant to consultant as to how woman centred they’re prepared to be. So you might find that somebody will agree something in advance … and then the consultant on the day is just not comfortable with it, the risk will have always been the same. What changes is the consultant who is there’* (Site B, Doula, 127)

Doulas highlighted that cervical dilation requirements had prevented pool use where women declined vaginal examination.

#### Use of equipment and resources

The way pool rooms were allocated on the obstetric unit in Site A enabled maximum use of the unit’s pools (see Table 3), for example through questions at triage and room changes.

*‘If women get out of the pool and want an epidural, we try and change the rooms, so that we can get the room cleaned and have it available for someone else, rather than have them sort of blocking the room’* (Site A, Obstetric unit, Band 5–6 Midwife, 214)

*‘You’ll hear the coordinator when she takes a phone call from triage … the question always is does she want to use the pool?’* (Site A, Obstetric unit, Band 7–8 Midwife, 202)

In Site B, although the pool room on the obstetric unit was kept free for women who wanted to use one, women tended to be automatically allocated a room without a pool. On the obstetric unit in Site C, the pool room was regularly used for women who did not wish to use the pool, so was rarely empty and room swapping was not facilitated.

*‘I think there is a culture of it’s kind of not the right place for waterbirths … it’s not facilitated for that reason, so if they’ve got somebody else in that room who doesn’t need it they wouldn’t swap them round for it’* (Site C, Obstetric unit, Band 5–6 Midwife, 405)

On the midwifery units in Sites B and C, the pool was typically filled when women arrived, which meant some women in advanced labour did not have the opportunity to use it. In Site A, the pool was routinely filled prior to women’s arrival, which midwives suggested encouraged pool use.

*‘They’ve got the choice, but we run the pool when they phone, it is ready and warmed up when they get here, and it just feels like a natural progression. Quite often they don’t even ask, or we don’t say do you want a waterbirth, they just get in’* (Site A, Midwifery unit, Band 7–8 Midwife, 305)

Pool rooms on the obstetric unit in Site A were considered more attractive than other birth rooms due to their size, decoration, and location, with one of the pool rooms located near to the midwives’ station, which was preferred by staff. In contrast, obstetric unit pool rooms in Sites B and C were less popular with midwives.

All midwives in Sites A and B were confident they could cope with emergencies in the pool. However, in Site C midwives based on the obstetric unit were anxious about emergency evacuation procedures and felt these were not well-practised.

*‘Hauling a woman out of the pool, when she’s wet and slippery … I’ve not had the training to use [the nets], so if I was in that situation I’d, I’d be unprepared … I haven’t looked after a woman in the pool. And I don’t think I’d want to … I wouldn’t feel equipped’* (Site C, Obstetric unit, Band 5–6 Midwife, 409)

Women wishing to use a pool at home in Sites A and B had to source their own equipment, which prevented some from using a pool for financial reasons. A community midwife in one site had purchased a pool to lend to women; however, this was not widely known. In contrast, in Site C, women wishing to use a pool at home were provided with a pool, pool liner and water pump, either by the Trust itself or in liaison with a local charity. This enabled all women who wished to, to have a home pool birth.

*‘I don’t think I’ve ever had a woman who’s really wanted a [home] waterbirth who’s not had the chance to have one’* (Site C, Community Midwife, 435)

#### Senior staff support for natural birth

In Sites A and B, obstetric consultants were supportive of the midwifery unit and women classed as low risk were encouraged to receive midwifery-led care, which in turn increased access to water immersion (see Table 3). In Site A, midwives were aware of support for the midwifery unit at all levels of management within the Trust, including the Chief Executive. Trust policy was encouraging of births in a non-obstetric setting, which were increasing. In Site B, women with complications who wanted to give birth on the midwifery-led unit could attend a midwifery-run clinic to discuss their birth plan.

*‘[Doctors are] definitely advocating birth centres more and more, which in turn then obviously does promote waterbirth essentially’* (Site A, Midwifery unit, Band 7–8 Midwife, 310)

Midwives and women in Site C felt that doctors tended to discourage women from accessing the midwifery unit, which impacted on pool use.

*‘They suspected that I had a blood condition … [but] when the bloods came back, they were … normal … every time and there was no reason for me to be induced [on the obstetric unit]. But the doctors were adamant. So they would say like, “She’s at risk of stillbirth” and stuff and things like that … But … the midwives were very, very helpful, they gave me the confidence to just carry on … ’* (Site C, Midwifery unit, Woman, 434)

*‘What it tends to end up as if you’re low risk you can use the pool, if you’re high risk you tend not to be able to, even if you would be suitable … any woman that ends up on labour ward tends not to end up in a pool, and in the birth centre it would be routine’* (Site C, Obstetric unit, Obstetrician, 401)

#### Women’s awareness of and attitudes towards pool use

In Sites A and C, women were invited to tour the midwifery unit and see a pool room. This was effective in encouraging pool use to be considered. In Site B, an online virtual tour was available (see Table 3). (It should be noted that due to restrictions related to Covid-19 at the time of writing, virtual tours of maternity units are now the norm).

*‘We’re lucky with the facilities at the birth centre, and everybody knowing about being able to come for a tour … Because then that prompts discussion about the pool, because people don’t usually expect the birth centre to be as nice as it is, because it’s gorgeous’* (Site C, Community Midwife, 420)

*‘When I fell pregnant I was straight away thinking epidural in hospital, but … I thought actually the birth centre was a much calmer, nicer place to give birth if I could’* (Site A, Midwifery unit, Woman, 319)

Community midwives at all sites highlighted that discussions about pool use were difficult to fit into antenatal appointments; therefore, antenatal classes were useful in raising awareness of this option. While in Site C, antenatal classes were available to all women, in Sites A and B they tended to be over-subscribed, so not all had the opportunity to attend.

*‘We don’t have [the] chance to discuss [pools] with every single woman … but everyone is told that they can come to the antenatal classes, and if they choose to come to them … then [pool use] … will be discussed with them at length’* (Site C, Community Midwife, 420)

*‘We didn’t get … to do [antenatal classes] … we were late booking them, so we didn’t get a chance to get in there’* (Site B, Midwifery unit, Woman, 121)

Site C serves some of the most deprived communities in the UK and has a significantly higher than average ethnic minority population; a factor that midwives felt may reduce waterbirth rates.

### Differences between obstetric and midwifery units

Differences in unit culture were more substantial between the obstetric and midwifery units within each site than between the same type of unit across the three case study sites. We identified ten main themes to describe these differences (see Table 4), broadly falling into the categories of physical environment; midwives’ intrapersonal factors; autonomy and support of midwives; and information and support for women. We explore each of these categories in turn, in relation to their impact on pool use.

**Table 4:** Key differences between obstetric and midwifery units

Categories and themes	Obstetric units	Midwifery units
*Physical environment*		
Pool availability	Pool in 7–17% of birth rooms	Pool in 60–100% of birth rooms
Pool room environment	Some pool rooms disliked by midwives	Described by women/midwives as relaxing/encouraging of pool use
*Midwives’ intrapersonal factors*		
Midwives’ attitudes	Some not keen on pool use, often due to lack of confidence	All very positive about pool use
Midwives’ confidence	Some not confident - frightened of pool use	All confident in supporting pool use
*Autonomy and support of midwives*		
Senior staff support	Some senior staff unsupportive of pool use	Midwives feel very supported by seniors to support pool use
Midwives’ autonomy	Some autonomy to offer the pool to ‘low risk’ women	Complete autonomy to offer the pool to all women
*Information and support for women*		
Women’s awareness	Women don’t necessarily know there is a pool, so don’t ask to use one	Women very aware of pools on the unit; most ask to use one
Information given to women	Pool use not fully discussed antenatally	Pool use discussed and encouraged antenatally
Proactive offering of pool	Pool not usually offered - women have to be proactive	Pool offered / promoted to all women
Support for women to use a pool	Pool use generally discouraged, and in some cases blocked	Women are supported / actively encouraged to use a pool

#### Physical environment

While the three midwifery units had sufficient pools to enable all women to use one if they wished to, there were only one or two pools in each of the obstetric units, which limited opportunities for water immersion.

*‘I have been on a couple of shifts where a lady’s wanted a pool, and they’ve both been taken up, and then she’s obviously had to not have the birth she was envisioning’* (Site A, Obstetric unit, Midwifery Support Worker, 211)

Some midwives suggested the number of pools available suited the low level of demand from women. However, others proposed that limited availability in itself affected requests to use a pool. Women felt there were insufficient pools on the obstetric units, and therefore believed accessing a pool would be unlikely. This meant they tended not to ask to use one, contributing to perceptions of low demand.

*‘In all honesty, there are actually very few women on here who do want to go in [the pool]’* (Site C, Obstetric unit, Band 7–8 Midwife, 408)

*‘There’s one pool in the whole [unit] and it is first come first served... I think I had that in my head … just even if I asked for it I probably wouldn’t get it’* (Site B, Obstetric unit, Woman, 117)

Women and midwives described the pool room environment on midwifery units as relaxing and encouraging of pool use. Although pool rooms on the obstetric units did provide an attractive environment for women (e.g. tending to be larger than other birth rooms with pictures or dimmed lighting), they appeared less popular with midwives in Sites B and C. For example, they were described as being located at the furthest point from the midwives’ station, set up differently to other birth rooms or not connected to the unit’s central fetal monitoring systems.

*‘With all the other rooms they are connected to [the remote monitoring system] so you know that you’ve kind of got eyes on that watching … You’re kind of on your own in the pool room, no one is watching it … ’* (Site B, Obstetric unit, Band 5-6 Midwife, 128)

*‘An unspoken thing … is that it’s not a very nice room, just in terms of its setup … It’s a bit more clinical in other rooms, so generally this room isn’t loved by midwives … It is just things are in different places … . I don’t know what’s in those drawers in order. Whereas if I went to any other room I would know’* (Site B, Obstetric unit, Band 5–6 Midwife, 119)

In the midwifery unit of Site A, pools were located at the centre of birth rooms with a couch to the side; midwives felt this made the pool the focal point of the room and the default option for women.

*‘When you walk into one of the delivery rooms, the most obvious point of the delivery room is the pool. And no bed. Often people say well where’s the bed?... I think that’s a really good trigger point and they often say oh that looks amazing, to get into a pool’* (Site A, Community Midwife, 302)

#### Midwives’ intrapersonal factors

Midwives working in midwifery units were unanimously positive about pool use and suggested this was part of the reason they had chosen to work in a midwifery-led setting.

*‘I love pool births and that’s half the reason I’m here. I think it just gives people immense pain relief, relaxation, and you just instantly see people relax as they get into it’* (Site A, Midwifery unit, Band 5-6 Midwife, 304)

*‘I think it’s all ingrained here that we’re very pro water and a lot of the staff will encourage it’* (Site B, Midwifery unit, Band 5–6 Midwife, 107)

In contrast, although some midwives working in obstetric units were positive about pool use, attitudes varied. They suggested this affected the likelihood of women being offered a pool.

*‘They wouldn’t offer it … If they’re not keen on pool births, they would go straight to Entonox, paracetamol, codeine, pethidine, epidural, rather than offering the pool’* (Site A, Obstetric unit, Band 5–6 Midwife, 203)

Midwives working in both obstetric and midwifery units mentioned physical issues related to supporting pool use, such as back problems. However, while this had not prevented any midwives based in midwifery units supporting women to use a pool, some midwives working in obstetric units did not attend pool births for this reason.

Due to their regular experience of facilitating waterbirth, midwives based in midwifery units were confident to support women in the pool.

*‘Coming across to a unit that [is] quite pro waterbirth it was just a confidence building thing for me and having had a good eight years I would say now of regular exposure to waterbirths, that’s really helped my confidence’* (Site A, Community Midwife, 302)

Waterbirth was a more unusual event on the obstetric units, particularly in Sites B and C that had obstetric-led birth rooms only; therefore, midwives working in obstetric units were less confident in - and in some cases frightened of - supporting women to use a pool.

*‘Some midwives are, are frightened … we’ve had midwives in other areas in tears, because they’re frightened of coming on here, haven’t … been witness or participated in a waterbirth, since … they were a student’* (Site C, Midwifery unit, Band 5–6 Midwife, 432)

*‘I certainly wouldn’t feel confident enough to be left in charge of a woman … delivering in the pool … I’ve just not got … the competence really … So I, I’d sort of defer and let somebody else … look after her really’* (Site C, Obstetric unit, Band 5–6 Midwife, 409)

It was suggested that rotations between obstetric and midwifery units could be instrumental in changing practice and increasing confidence in waterbirth.

*‘When midwives are really confident in high risk … their high-risk care, starts to drip into the midwifery-led [unit], transfer rates go up, intervention rates start to go up. Whereas if you see it the other way, their normal care starts to get infiltrated into the women [on the obstetric unit]. So you see a peak in the pool being used [on the obstetric unit], because it’s a midwife that’s really confident with waterbirth’* (Site B, Band 7–8 Midwife, 102)

Midwives proposed they and their colleagues fitted into one of two categories: ‘low risk’ midwives who are confident in low risk settings, and ‘high risk’ midwives who are skilled at supporting higher risk women but less comfortable with low risk births.

*‘Some midwives don’t like working in the midwifery-led unit, they’re not midwifery-led midwives, they’ll call themselves high risk midwives … they will be then the midwives that are highly skilled in looking after that diabetic mother, or you know, all of those kind of things, and their knowledge in a different area will be absolutely superb and much more superior to somebody else, but switch their roles, and they both feel really uncomfortable and out of their comfort zone’* (Site B, Band 7–8 Midwife, 102)

#### Autonomy and support of midwives

Midwives based in midwifery units felt supported and encouraged by senior midwifery staff to facilitate water immersion and were expected to offer the pool to women. In the obstetric units, some obstetric consultants and senior midwives were described as being supportive of pool use, while others were more averse. Pool use was seen by some as being suitable only for low risk women and not appropriate for an obstetric unit.

*‘I had a lady that asked for a waterbirth and was told no. Even though I kind of said but she is low risk … I was told categorically no … A lot of the time because I know certain staff [are] on … I wouldn’t offer [the pool because I know] they wouldn’t allow it … which is sad really’* (Site C, Obstetric unit, Band 5–6 Midwife, 405)

*‘We fought hard to get a pool on the labour ward … I think some of the consultants wanted twelve high risk rooms’* (Site C, Midwifery unit, Band 7–8 Midwife, 428)

*‘I think [a pool is] something that birth centres should have. I am not sure about it being on labour ward because we get the more complicated people, and you want to monitor them more’* (Site C, Obstetric unit, Obstetrician, 416)

Whereas women giving birth on the midwifery units were seen as being pre-approved to use a pool, on obstetric units, midwives had to use their own judgement, which could leave them open to criticism. While some obstetric consultants viewed waterbirth as safe, midwives suggested some doctors and senior midwives were quick to attribute any problems to pool use. Therefore, some midwives working on obstetric units feared they could be blamed if an adverse outcome occurred.

*‘Everybody’s happy if the outcome is good, but if [it’s not] … they go back and back and back … and then they’ll start looking and saying so you know, her blood pressure was up once, so why did you put her in the pool? So that is sometimes the issue … It’s not quite as black and white up here to put somebody in the pool as it is on the midwifery-led unit’* (Site B, Obstetric unit, Band 7–8 Midwife, 133)

*‘I can think of … a waterbirth and the baby was six hours old. Everything was nice and straightforward until we noticed that the baby was grunting … actually we weren’t overly concerned … [But] the paediatrician … said it was because it was a waterbirth … actually there’s no research that supports that’* (Site A, Midwifery unit, Band 7–8 Midwife, 310)

Midwives working in midwifery units considered they had complete autonomy to offer the pool to women, whereas midwives in obstetric units were more likely to check with senior staff before facilitating pool use.

*‘Women are down here because things are normal so you can just say “Fine” and turn the taps on … you don’t have to, you might update your, your senior but at that point you can make the decision’* (Site B, Midwifery unit, Band 5–6 Midwife, 106)

*‘I think [on the obstetric unit] you [need to check with senior staff] because the women’s cases are complex … If I compare to my [midwifery-led] placement … [it] was just a standard thing, oh you can have the pool. Up here … well as much as you are autonomous you are and you’re not really. You’re forever double checking nearly every decision you make … So I don’t feel like really with anybody that I would just say yeah let’s go and use the pool’* (Site B, Obstetric unit, Band 5–6 Midwife, 130)

#### Information and support for women

Women receiving midwifery-led care were usually aware of the option to use a pool, having been informed through antenatal classes, discussions with midwives or tours of the unit. As water immersion was actively promoted, women who had not previously thought about using a pool were encouraged to consider this option. In contrast, for those receiving obstetric-led care, midwives and women reported that pool use was discussed little or not at all during the antenatal period.

*‘When I first found out that I was pregnant, [waterbirth] was something that I … didn’t want to consider, I didn’t want to be in water and be wet … but once I’d been to the antenatal … it was something that changed my mind and something I would definitely want to do’* (Site C, Midwifery unit, Woman 404)

*‘Once I was told I was moved to being consultant-led, I was under the impression that a waterbirth wouldn’t necessarily be something that I could consider … . As the pregnancy went on it just became something I thought is probably not going to happen’* (Site B, Obstetric unit, Woman, 117)

*‘I think if it’s high risk, I don’t think [pool use is] discussed … it just gets dropped … and I don’t think it is given as a valid, potential option … If your obstetric staff are not keen on it, it won’t be promoted via them, they’ll actually try and put women off, and if the midwives are not experienced in it, then they won’t promote it either’* (Site C, Obstetric unit, Band 7–8 Midwife, 402)

Similarly, during labour the pool was offered routinely to women giving birth in the midwifery units, whether or not it was requested. Women felt encouraged to try using water immersion, even where they had not considered it antenatally. On the obstetric units, water immersion was not seen as a primary option for analgesia and was often not considered by midwives as part of routine care, particularly where they were not keen on pool use. Therefore, the pool tended to be requested rather than offered, and midwives felt that women would need to be proactive to gain access to water immersion. Where women did request to use a pool on the obstetric units, they were sometimes discouraged or blocked from doing so.

‘*It seems to me … that [the pools are] not always offered … even if they are available, as a first line. If you were in the birthing centre they’d be filling the pool practically before you got there. Whereas [on the obstetric unit] … it’s an afterthought’* (Site A, Community Midwife, 217)

*‘I’ve never seen it be offered … The only time I’ve ever seen a woman in the pool [on the obstetric unit] was because she requested it’* (Site B, Midwifery unit, Student Midwife, 109)

*‘In the hospital you … hear people telling [women] that a pool is not available because they don’t want to use them’* (Site A, Midwifery unit, Band 5–6 Midwife, 304)

## Discussion

This case study research provides new insights into the influence of maternity unit culture on pool use, through examination of the differences between six UK maternity units with varying waterbirth rates. Consistent with UK pool use statistics [27] and previous qualitative research [30], the greatest differences in unit culture were between obstetric and midwifery units within case study sites, rather than across the sites themselves. This suggests it is the underpinning model of care that is most significant in influencing water immersion practices, rather than unit-level factors. However, the differences between sites highlight more nuanced differences in approach that can also act as barriers to or facilitators of pool use. Criteria for pool use, equipment and resources, staff attitudes and confidence, senior staff support for colleagues and endorsement of physiological birth were identified as key organisational factors, alongside information provided and encouragement of women to use the pool. Findings suggest unit culture is of crucial importance and indicate changes to practice that could improve access to water immersion.

Building on our previous research [30], we found pool availability had a broad impact, affecting women’s choice of maternity unit, requests for water immersion during labour, midwives’ ability to offer the pool and confidence in relation to pool use. Although limited pool availability acted to reduce demand for water immersion, low demand was often cited as a reason for limiting the number of pools available, creating a vicious circle in which pool provision remained low. As found by Russell [28, 29] and Milosevic et al. [30], birth room allocation practices were a key influence on pool access and appeared to reflect the extent to which pool use was supported by the maternity unit. We also found that the timing of filling the pool influenced the extent to which women were encouraged or enabled to use water immersion.

It is not just pool availability that is important. Environment matters too, with midwifery units providing welcoming pool rooms, while obstetric unit pool rooms had barriers to use such as poorer monitoring facilities. Although several studies [36–38] show that birth room environment positively influences labour experience and outcomes, how this occurs, and the implicit messages conveyed by environment, have not been fully explored.

The relationship between midwives’ attitudes to water immersion, their confidence in offering the pool to women, and the setting is a complex one. In terms of encouraging water immersion, there was a virtuous circle in the midwifery units of midwives being confident and experienced, with easier access to facilities, and supported by senior staff. Conversely, in the obstetric units, midwives were often fearful of facilitating pool use, had fewer opportunities to gain experience, and worked with senior staff who themselves did not support water immersion or promote the pool as an option. This supports the assertion of Nicholls et al. [39] that confidence in facilitating natural birth is developed through midwives’ exposure to settings where physiological birth is the dominant culture. In the present study, midwives highlighted fundamental differences in the beliefs and competences of those working in the different settings. It is not possible to elicit the causal chain in this situation: whether midwives are attracted to a certain type of setting due to previous preference, or whether finding themselves in a particular setting they develop the beliefs, competences and confidence to match, conforming to the norm for that setting. These differences and this interplay of factors have the potential to impact considerably on the way care is delivered, and thus merit further exploration.

Water immersion was described as being ‘pre-approved’ and offered routinely as part of standard care on midwifery units, so midwives had full autonomy to offer the pool. In the obstetric settings, a medicalised, risk-averse approach was normalised, with all labours and births conceptualised as ‘high-risk’, thus needing to be managed via medical surveillance. Midwives incorporated this description into their identity, describing themselves (or others) as a ‘high risk midwife’. On obstetric units, water immersion was usually precluded except when clearly requested by women (as also found by Russell [28, 29]). If midwives wanted to offer the pool, they were not always supported by medical staff, some of whom believed it was not appropriate in a high-risk setting (as found by Ulfsdottir et al. [20]), regardless of individual circumstances.

Lack of training and unfamiliarity with equipment were both given as reasons for midwives based in obstetric units being unable or reluctant to care for women in a pool. Such attitudes would not be tolerated towards other equipment on obstetric units, suggesting an acceptance among all levels of staff that the option for women to use a pool on an obstetric unit is viewed as an optional luxury rather than an essential midwifery skill.

Over 80% of births in the UK occur in obstetric units [26]. Even taking account of intrapartum transfers from midwifery to obstetric units, this suggests many women who could safely plan to labour in midwifery-led settings are currently not doing so. There is known inconsistency in admission criteria for midwifery units [40]; more individualised assessment of risk by midwives and obstetricians is required if women who are appropriate for midwifery care are to be identified and encouraged to consider this option. This is an area where more process research is needed; having reliable data on the safety of waterbirth will be a key component in addressing the challenge of intervention reduction.

The existence of guidelines should have the potential to support midwives’ confidence and autonomy. However, we found that inconsistent implementation across case study sites meant some women were prevented from using a pool when trust/health board policy would have supported them to do so. Pool use guidelines at all three sites had relaxed in recent times, with a general shift towards assessing suitability on an individualised basis; for example increasing cut-off BMIs, removing cervical dilation requirements, and allowing women who are carriers of Group B Strep to use a pool. Midwives generally welcomed this increased flexibility; however not all were aware of the changes.

As found in this research, evidence suggests antenatal information plays a vital role in enabling women to make an informed choice about water immersion [24, 41, 42]. It is particularly important in starting to democratise pool use and break down some of the barriers, either real or assumed, to women from different backgrounds accessing waterbirth.

### Recommendations for practice

Midwives, doctors and managers working within obstetric-led care need to be aware of potential barriers to water immersion so they can act to reduce these and support use of pools where appropriate. Increasing exposure to waterbirth for student midwives and midwives in obstetric units may enhance confidence and be instrumental in changing unit culture. Strong midwifery leadership, for example through ensuring the presence of a consultant midwife in all settings (as recommended by the Royal College of Obstetricians and Gynaecologists [43]), is also key to ensuring a culture of normal labour and birth is supported and facilitated.

Providing obstetricians and neonatologists with information on and exposure to waterbirth could increase support for pool use, reduce adverse events being erroneously attributed to water immersion and increase confidence in responding to emergencies in the pool. Applying waterbirth guidelines consistently will help ensure that women who can safely use a pool are enabled to do so.

Even in areas with perceived low demand for water immersion, increasing the provision of pools in birth rooms can enhance midwives’ experience and confidence, enable water immersion to be offered more frequently and encourage women to request to use a pool. Birth room allocation practices should also be considered, to facilitate more effective use of unit resources. Thought should be given to the birth room environment, to ensure all birth rooms are attractive to both women and midwives, and that the layout of pool rooms encourages use of water immersion. Filling the pool ready for a woman’s arrival may also facilitate this.

More widely, it is vital that in the antenatal period women are provided with the information they need to make an informed choice about pool use. Time constraints may make it difficult to discuss this in routine antenatal appointments, so it is important that antenatal information and/or maternity unit tours are available for all. At an organisational level, support for midwifery-led units and home birth can help increase access to these settings, and in turn, pool use.

### Strengths and limitations

Taking a case study approach enabled an in-depth exploration of factors influencing water immersion, through the inclusion of multiple perspectives and data sources. Situating the research in six maternity units allowed for the examination of the research issue in a real-world context, providing a rich insight into unit culture.

Case study sites were purposively selected to encompass maternity units with a range of waterbirth rates, and the study sample was large and diverse, enhancing generalisability. However, units included in the study were part of a self-selecting group of NHS organisations opting to participate in the POOL study. Therefore, they were all research-active and facilitated at least some waterbirths, which is likely to have affected findings.

Despite utilising a range of recruitment strategies, we experienced difficulties recruiting particular participant groups (doulas, postnatal women and doctors). Subsequently, study recruitment was curtailed prematurely due to restrictions related to Covid-19, so these viewpoints were under-represented in some sites. Nevertheless, the range of data captured from each case study unit allowed for comprehensive examination of the research issue.

### Implications for future research

Future research could examine how pool availability impacts on demand for water immersion, and how midwives’ attitudes influence their access to training and familiarity with equipment. Building on previous studies [36–38], the effect of the birth room environment on pool use could be investigated. In addition, while water immersion guidelines themselves have been extensively examined [21, 22, 44, 45], further exploration of their implementation in practice is needed.

It is well-established that antenatal discussions play an instrumental role in ensuring women are fully informed about pool use [24, 41, 42]. Building on this, it would be useful to examine the relative value of different modes of information delivery; for example, comparing online information, antenatal appointments, classes (NHS vs. private) and maternity unit tours. As more services have shifted to being delivered online due to Covid-19, it would be interesting to explore whether this will provide more democratic provision of antenatal information.

Further research investigating the inter-relationship between the attitudes and competences of midwives who choose to work in obstetric or midwifery settings, and the practices and culture of the settings themselves, will be crucial to understanding the differences in the way maternity care is provided. This would have implications for pre- and post- registration training, resourcing and ongoing staff development.

## Conclusions

Maternity unit culture has a substantial influence on pool use. We found considerable differences between obstetric and midwifery units in relation to equipment and resources, staff attitudes and confidence, senior staff support and women’s awareness of water immersion. Findings have several implications for practice: increased exposure to waterbirth is vital to improve the confidence of midwives working in obstetric units; training for obstetricians and neonatologists on the practicalities of pool use could increase support for water immersion; and improved access to antenatal information would help increase awareness of the option to use a pool. We recommend that obstetric units increase pool provision, ensure birth room allocation maximises the use of unit resources, design pool room environments that encourage use, and ensure all midwives take responsibility for being familiar with water immersion equipment and unit guidance.

## Supplementary Information

**Additional file 1 Interview Topic Guide - Band 5–6 Unit Midwives**.

**Additional file 2 Interview Topic Guide - Band 7–8 Unit Midwives**.

**Additional file 3 Interview Topic Guide - Community Midwives**.

**Additional file 4 Interview Topic Guide – Doulas**.

**Additional file 5 Interview Topic Guide – Midwifery Support Workers**.

**Additional file 6 Interview Topic Guide – Obstetricians and Neonatologists**.

**Additional file 7 Interview Topic Guide – Postnatal Women**.

**Additional file 8 Interview Topic Guide – Student Midwives**.

## Data Availability

The datasets generated and analysed during the current study are not publicly available and cannot be shared as individual privacy could be compromised if full interview transcripts were released.

## Abbreviations

UK: United Kingdom
NHS: National Health Service
MU: Midwifery unit
OU: Obstetric unit
