# Supplementary material for: Factors influencing water immersion during labour: qualitative case studies of six maternity units in the United Kingdom
Source: BMC Pregnancy Childbirth. 2020 Nov 23;20:719. doi: 10.1186/s12884-020-03416-7 (PMC7682119; doi:10.1186/s12884-020-03416-7)
Supplement: Supplementary file 1 — Additional file 1 Interview Topic Guide - Band 5–6 Unit Midwives. [file 12884_2020_3416_MOESM1_ESM.docx]

**Interview Topic Guide - Band 5-6 Unit Midwives**

**Introduction**

- Thank participant for agreeing to take part
- Are you happy for our conversation to be recorded for transcription and analysis?
- Start audio-recording
- The aim of this discussion is to explore your experiences and opinions in relation to the use of birth pools generally, and particularly to focus on the use of birth pools in this unit.
- What we talk about today will be kept confidential – only members of the research team will have access to the recording, and it won’t be labelled with your name. We might use some quotes from discussions in publications or presentations, but no names will be used.
- The aim of the discussion is to find out about your views and experiences, so there are no right or wrong answers.
- If there are any questions you don’t want to answer or if you would like to stop the recording or leave at any time, please let me know.
- Would you like to ask any questions before we start?

**__________________________________________________________________________________**

**Views of pool use for labour and birth**

- What is your view of women using a pool for labour and birth? / Do you think it is a good or a bad thing?
- Do you like supporting women who are having a waterbirth or labouring in water?
- Do you prefer it if women get out of the pool to deliver?
- Do some women prefer to get out of the pool to deliver?
- Can you see any benefits of waterbirth or using a pool during labour?
- Do you think there are any negative aspects or risks?
- Do you have any concerns about the safety of waterbirth or using a pool during labour?
- Do you think there should be more or fewer waterbirths on the unit? Why?
- How does pool use affect your day-to-day work?
- Are waterbirths harder work or more difficult for you than births on dry land?
- In terms of monitoring / delivery / physically?
- Do you think that waterbirth gives you as a midwife less control?
- How do you think waterbirth and using a pool during labour is viewed by staff on the unit?
- By midwives?
- By midwifery managers?
- By consultants?
- Is waterbirth ever discussed on the unit?
- Do the risks or benefits of waterbirth tend to dominate discussions?
- Do some staff regard waterbirth as an added risk with no value?
- Do all staff view waterbirth in the same way, or do you think different members of the team have different views of waterbirth?
- Are there any waterbirth ‘champions’ on the team?
- Is there anyone particularly against waterbirth?
- Do you think that offering a pool to women has any impact on relationships with your colleagues?
- Do some midwives facilitate more waterbirths than others? Is there a difference between more experienced and newer midwives?
- Is using a pool during labour seen as being part of routine care or as being unusual?
- Has the way waterbirth is viewed on the unit changed over the last few years?
- How much autonomy do you have in regards to pool use and waterbirth?
- Who makes the decisions as to whether a woman uses a pool or gives birth in water?
- When women can get in the pool? / If/when they have to get out?
- Do you think there is support from consultants for pool use?
- Can they see any benefits of pool use?
- Do they prefer women to get out of the pool to deliver?
- Do they have any concerns about the safety of waterbirth or use of a pool in labour?
- What is the main message about waterbirth that staff hear from the senior team?
- Do you feel supported to support women to use a pool or give birth in water?
- What about women classified as ‘high risk’?
- Generally, do you feel that women can have a waterbirth on the unit if they wish?
- Is there anything that stops you offering waterbirth as a choice?
- Do you feel you can advocate for women even where their choices go against what consultants want?
- Have you ever felt unable to support a woman’s choice of waterbirth? (If yes) – What happened?
- Do you think that pool use for labour and birth is more likely when women give birth at home? Why?

**Views of non-medicalised birth**

- Do you feel that birth is over- or under-medicalised on the unit? (e.g. that there is too much/ too little monitoring or intervention)
- What is the usual level of monitoring in labour?
- For high risk / low risk women?
- Continuous or intermittent monitoring? How often?
- Do you feel supported to support women to have a natural birth?
- Are natural births seen as the norm on the unit?
- Is there consultant support for non-medicalised birth?
- How much do you think consultants know about natural birth?
- What do you think their views on natural birth are?
- Do they see any benefits / disadvantages?

**Staff confidence, knowledge and experience**

- How experienced are you in facilitating waterbirth? / How frequently do you support women having a waterbirth or labouring in water?
- How knowledgeable do you feel about waterbirth?
- What training have you had in relation to waterbirth? Did you find this useful? Is there anything you feel you would like more training on?
- How confident do you feel about supporting women having a waterbirth or labouring in water?
- Would you feel confident in coping with emergencies in the pool?
- What do you think has helped you to be confident about facilitating waterbirth? / Why do you think you’re not confident about facilitating waterbirth? What would help you be more confident/ knowledgeable?

**Women’s awareness of pool use as an option for labour and birth**

- Do women tend to be aware of the option to use a pool before they arrive at the unit in labour?
- How do they find out about it?
- What expectations do women have about waterbirth on the unit?
- Do women tend to be aware of the option to use a pool once they have been admitted to the unit in labour?
- How visible are the pools on the unit? Is there one in every delivery room?
- What information are women given in pregnancy about the unit?
- Do they have a leaflet about the unit? Does it include pictures/information about pools?
- Do they have a tour of the unit? Would this include seeing a birth pool?
- What do women generally know about waterbirth?
- What concerns or questions do they raise about waterbirth?
- Do they have any misconceptions about waterbirth?
- Do you generally encourage women to try using a pool for labour and/or birth, or do you tend to offer pools only to women who request one?
- (If encourage): When offering a pool, what do you say to encourage women to try using it?
- Is there anything that would affect how likely you are to suggest using a pool to women?
- How proactive do you think women have to be to have a waterbirth on the unit?
- Roughly what proportion of women do you think request to use a pool? Why do you think this is?
- If women request to use a pool, are they usually able to?
- Are there certain groups of women who are more or less likely to request to use a pool?
- Are there certain groups of women who are more or less likely to get access to a pool?

**Criteria for pool use and how these are applied**

- Are there any unit policies, procedures or guidelines to follow relating to pool use?
- What are the criteria for women being allowed to use a pool on the unit? / Are there any groups of women who are not allowed to use a pool? Why?
- Are there any groups of women who are only allowed to use a pool under certain conditions (e.g. monitoring/leaving pool prior to birth)? Why?
- Can high risk women use a pool? How is ‘high risk’ defined?
- Is there a cut-off BMI for pool use? Why?
- Can women who need monitoring use a pool?
- Do you know what the criteria are for women being allowed to use a pool at home? Is this different to the criteria for pool use on the unit? (If so, why?)
- Are there any unit guidelines relating to when women can get into the pool? (e.g. when x cm dilated)
- Are there any unit guidelines relating to women having to get out of the pool or not deliver in water in certain circumstances? (e.g. in the case of certain complications)
- Are there any ‘unwritten’ policies, procedures or guidelines relating to pool use on the unit?
- Does what happens in practice on the unit tend to stick to the guidelines, or do staff sometimes tweak them?
- Can any of the guidelines be overruled? (e.g. by women’s choice / in certain cases / by certain staff)
- What are the criteria for transfer to consultant-led care?
- What are the unit policies, procedures or guidelines relating to monitoring?
- Is continuous monitoring required in all/certain cases?
- How helpful do you think the unit policies, procedures or guidelines are?
- Are there any problems with them? (e.g. are they too restrictive/inflexible?)
- Do you think that unit policies, procedures and guidelines support and encourage waterbirth? Why/why not? Why do you think that is?
- Do you know who it is that sets the guidelines, or how they have been decided upon?
- (If there have been any rises/falls in waterbirth rates on the unit) – Why do you think this is?

**Equipment and resources**

- How many pools do you have on the unit?
- How many are in working order and can be used?
- (If applicable) – What is the problem with the pool/s that can’t be used?
- Are there enough pools? Can women who want to use one always do so?
- (If few pools) – Why is this?
- How long does it take to get a pool ready for the next patient after it has been used?
- What is the process?
- Who does this?
- Are there any issues with this?
- How long does it take to fill the pool?
- Has the length of time the pool takes to fill prevented some women from using it?
- At what point is the pool filled? (e.g. when the woman phones in, when they are x cm dilated, etc.)
- Are there any technical issues with using the pool? (e.g. temperature issues, etc.)
- Are there any physical issues with supporting women who are using the pool? (e.g. bad backs, etc.)
- Are there enough staff on the unit?
- Does staffing impact on pool use?
- Are there enough staff trained and experienced in waterbirth?
- Are all staff trained and experienced in waterbirth? How many are/aren’t? Why is this?
- Are there several different types of pool on the unit? (e.g. some inflatable)
- (If yes) – Which type do you prefer / not like? Why?
- Are there any technical issues with any of them?
- Does the unit have waterproof monitoring equipment?
- Is this readily available? (If no) – Why?
- Are there any technical issues with it?
- Does it work in all rooms with a pool?
- What are the rooms with pools like?
- How do they compare to the rooms without pools?
- If pool rooms are ‘nicer’, are they sometimes used for women who don’t use the pool?
- Where are the pools located?
- Does the location of the pools cause any issues?
- Do you think pools are used efficiently on the unit?
- Are there often pools that aren’t being used? Why do you think this is?
- Are there sometimes women in rooms with pools waiting for discharge, etc.?
- Could pool use be increased with better management of resources?
- What do you advise women about pool availability?
- Do you feel you can promote using a pool to women, or would you be concerned that you might encourage them to do this when there might not be a pool available?
- Are there any other issues relating to equipment and resources for pool use?

__________________________________________________________________________________

**End of interview**

- We’ve covered all of my questions – is there anything that we haven’t mentioned that you would like to say about the use of birth pools?
- Thank you for taking the time to talk to me today.
- Stop audio-recording.
