# Supplementary material for: Factors influencing water immersion during labour: qualitative case studies of six maternity units in the United Kingdom
Source: BMC Pregnancy Childbirth. 2020 Nov 23;20:719. doi: 10.1186/s12884-020-03416-7 (PMC7682119; doi:10.1186/s12884-020-03416-7)
Supplement: Supplementary file 3 — Additional file 3 Interview Topic Guide - Community Midwives. [file 12884_2020_3416_MOESM3_ESM.docx]

**Interview Topic Guide - Community Midwives**

**Introduction**

- Thank participant for agreeing to take part
- Are you happy for our conversation to be recorded for transcription and analysis?
- Start audio-recording
- The aim of this discussion is to explore your experiences and opinions in relation to the use of birth pools generally, and particularly to focus on the use of birth pools in local maternity services.
- What we talk about today will be kept confidential – only members of the research team will have access to the recording, and it won’t be labelled with your name. We might use some quotes from discussions in publications or presentations, but no names will be used.
- The aim of the discussion is to find out about your views and experiences, so there are no right or wrong answers.
- If there are any questions you don’t want to answer or if you would like to stop the recording or leave at any time, please let me know.
- Would you like to ask any questions before we start?

__________________________________________________________________________________

**Views of pool use for labour and birth**

- What is your view of women using a pool for labour and birth? / Do you think it is a good or a bad thing?
- Do you like supporting women who are having a waterbirth or labouring in water?
- Do you prefer it if women get out of the pool to deliver?
- Do some women prefer to get out of the pool to deliver?
- Can you see any benefits of waterbirth or using a pool during labour?
- Do you think there are any negative aspects or risks?
- Do you have any concerns about the safety of waterbirth or using a pool during labour?
- Do you think there should be more or fewer waterbirths? Why?
- How does pool use affect your day-to-day work?
- Are waterbirths harder work or more difficult for you than births on dry land?
- In terms of monitoring / delivery / physically?
- Do you think that waterbirth gives you as a midwife less control?
- How do you think waterbirth and using a pool during labour is viewed by midwifery staff?
- By midwifery managers?
- Consultants?
- Is waterbirth ever discussed amongst your colleagues?
- Do the risks or benefits of waterbirth tend to dominate discussions?
- Do some staff regard waterbirth as an added risk with no value?
- Do all staff view waterbirth in the same way, or do you think different members of the team have different views of waterbirth?
- Are there any waterbirth ‘champions’ on the team?
- Is there anyone particularly against waterbirth?
- Do you think that offering a pool to women has any impact on relationships with your colleagues?
- Is using a pool during labour seen as being part of routine care or as being unusual?
- Has the way waterbirth is viewed amongst your colleagues changed over the last few years?
- Who makes the decision as to whether a woman uses a pool or gives birth in water?
- What is the main message about waterbirth that staff hear from the senior team?
- Do you feel supported to use a pool or give birth in water?
- Generally, do you feel that women are supported to have a waterbirth if they wish?
- Have you ever felt unable to support a women’s choice of waterbirth? (If yes) – What happened?
- Do you think that pool use for labour and birth is more likely when women give birth at home? Why?

**Staff confidence, knowledge and experience**

- How experienced are you in facilitating waterbirth? / How frequently do you support women having a waterbirth or labouring in water?
- How knowledgeable do you feel about waterbirth?
- What training have you had in relation to waterbirth? Did you find this useful? Is there anything you feel you would like more training on?
- How confident do you feel about supporting women having a waterbirth or labouring in water?
- Would you feel confident in coping with emergencies in the pool?
- What do you think has helped you to be confident about facilitating waterbirth? / Why do you think you’re not confident about facilitating waterbirth? What would help you be more confident/knowledgeable?

**Women’s awareness of pool use as an option for labour and birth**

- Do women tend to be aware of the option to use a pool?
- How do they find out about it?
- What do women generally know about waterbirth?
- What concerns or questions do they raise about waterbirth?
- Do they have any misconceptions about waterbirth?
- What information is provided to women antenatally about waterbirth and options available?
- How much time do you spend discussing birth options with women? What information do you give them?
- Do you know if there are any differences in the information provided by NHS and private antenatal classes, in relation to pool use?
- Are women having home births provided with different/more/less information about waterbirth as an option?
- How proactive do you think women have to be to find out about waterbirth?
- How proactive do you think women need to be to have a waterbirth?
- Roughly what proportion of women do you think use a pool at home? Why do you think this is?
- Are there certain groups of women who are more or less likely to use a pool?
- Are there certain groups of women who are more or less likely to have a home birth?

**Criteria for pool use and how these are applied**

- Are there any local policies, procedures or guidelines to follow relating to pool use?
- Are there any groups of women who are not allowed to use a pool? Why?
- Are there any groups of women who are only allowed to use a pool under certain conditions? (e.g. monitoring/leaving pool prior to giving birth) Why?
- Are there any local guidelines relating to when women should get into the pool? (e.g. when x cm dilated)
- Are there any local guidelines relating to women having to get out of the pool or not deliver in water in certain circumstances? (e.g. in the case of certain complications)
- Are there any ‘unwritten’ policies, procedures or guidelines relating to pool use?
- Does what happens in practice tend to stick to the guidelines, or do staff sometimes tweak them?
- Can any of the guidelines be overruled? (e.g. by women’s choice / in certain cases / by certain staff)
- What are the criteria for transfer to consultant-led care?
- Are there any policies, procedures or guidelines for you to follow in relation to monitoring?
- Is continuous monitoring required in all/certain cases?
- Can women who need continuous monitoring use a pool?
- How helpful do you think the policies, procedures or guidelines you have to follow are?
- Are there any problems with them? (e.g. are they too restrictive/inflexible?)
- Do you think that the policies, procedures and guidelines support and encourage waterbirth? Why/why not? Why do you think that is?
- Do you know who it is that sets the guidelines, or how they have been decided upon?

**Equipment and resources**

- Are all community staff trained and experienced in waterbirth?
- How many are/aren’t? Why is this?
- Do you have waterproof monitoring equipment available in the community?
- Is this readily available? (If no) – Why?
- Are there any technical issues with it?
- Does it work in all settings?
- Do you know if there are instances where women have a home birth and hire a pool, in order to guarantee they’ll be able to use a pool in labour? (e.g. in response to concerns about pool availability on the unit)
- Do you know if there are some women who would like to do this but can’t? Why can’t they? (e.g. cost/availability/home circumstances)
- Is there an NHS (or private) pool rental service for home waterbirths?
- How is this advertised?
- How popular is it?
- Are there ever any issues with pool availability?
- What is the cost?
- Are there any other issues relating to equipment and resources for pool use?

__________________________________________________________________________________

**End of interview**

- We’ve covered all of my questions – is there anything that we haven’t mentioned that you would like to say about the use of birth pools?
- Thank you for taking the time to talk to me today.
- Stop audio-recording.
