# Supplementary material for: Factors influencing water immersion during labour: qualitative case studies of six maternity units in the United Kingdom
Source: BMC Pregnancy Childbirth. 2020 Nov 23;20:719. doi: 10.1186/s12884-020-03416-7 (PMC7682119; doi:10.1186/s12884-020-03416-7)
Supplement: Supplementary file 4 — Additional file 4 Interview Topic Guide – Doulas. [file 12884_2020_3416_MOESM4_ESM.docx]

**Interview Topic Guide – Doulas**

**Introduction**

- Thank participant for agreeing to take part
- Are you happy for our conversation to be recorded for transcription and analysis?
- Start audio-recording
- The aim of this discussion is to explore your experiences and opinions in relation to the use of birth pools generally, and particularly to focus on the use of birth pools in this unit / local maternity services.
- What we talk about today will be kept confidential – only members of the research team will have access to the recording, and it won’t be labelled with your name. We might use some quotes from discussions in publications or presentations, but no names will be used.
- The aim of the discussion is to find out about your views and experiences, so there are no right or wrong answers.
- If there are any questions you don’t want to answer or if you would like to stop the recording or leave at any time, please let me know.
- Would you like to ask any questions before we start?

__________________________________________________________________________________

**Views of pool use for labour and birth**

- How do you think waterbirth is viewed by staff on the unit?
- Do you think this view has changed over the last few years?
- Do you think that all staff on the unit view waterbirth in the same way, or do different members of the team have different views of waterbirth?
- Are there any waterbirth ‘champions’?
- Do you know if there is anyone who is particularly against waterbirth?
- Do you think some staff regard waterbirth as an added risk with no value?
- Do you think waterbirths are seen as being part of routine care or as being unusual?
- By staff on the unit?
- By women?
- What is your view of waterbirth?
- Do you think there are any benefits?
- Do you think there are any negative aspects or risks?
- Do you like attending waterbirths?
- Are waterbirths harder work or more difficult for you than births on dry land? In terms of monitoring? Delivery? Physically?
- Do you prefer it if women get out of the pool to deliver?
- Do some women prefer to get out of the pool to deliver?
- Do you think waterbirth is more likely when women give birth at home? Why?

**Views of non-medicalised birth**

- Do you feel that birth is over- or under-medicalised on the unit? (e.g. that there is too much/ too little monitoring or intervention)
- Do you think natural births are seen as the norm on the unit?
- What is the usual level of monitoring in labour?
- For high risk / low risk women?
- Continuous or intermittent? How often?

**Staff confidence, knowledge and experience**

- How confident do you think midwives on the unit are in supporting women to use a pool and give birth in water?
- Do you think women feel supported to have a waterbirth?
- Do you think that women giving birth on the unit can have a waterbirth if they wish?
- Are there any examples you can think of where women have been prevented from having a waterbirth or using a pool during labour?

**Women’s awareness of pool use as an option for labour and birth**

- How do women in this area find out about waterbirth?
- Do you know what information is provided to women antenatally about waterbirth and options available?
- Do you know if there are any differences in the information provided in NHS and private antenatal classes, in relation to pool use?
- Do you know if women having a home birth are provided with different/more/less information about waterbirth as an option?
- How proactive do you think women have to be to find out about waterbirth?
- What do you think local women know about waterbirth?
- Are you aware of any misconceptions that some women have about waterbirth?
- What concerns or questions do women raise about waterbirth?
- What expectations do you think women have about waterbirth on the unit?
- Do you know how visible pools on the unit are? Do you know whether all the delivery rooms have one?
- Do you think women are encouraged to use a pool on the unit?
- Do you know if pools are offered proactively by midwives, or only on request?
- Do you know if there is anything that might affect the extent to which pools are promoted as an option to women (both prior to and during labour)?
- How proactive do you think women have to be to have a waterbirth?
- Do you think that certain groups of women are more likely to request a waterbirth?
- Are certain groups of women more likely to get access to a waterbirth?

**Criteria for pool use and how these are applied**

- Do you know if there are any groups of women who are not allowed to use a pool? Why?
- Do you know if there are any groups of women who are only allowed to use a pool under certain conditions (e.g. monitoring/leaving pool prior to giving birth)? Why?
- Do you know if there are any unit policies relating to when women can get into the pool? (e.g. when x cm dilated)?
- Do you know if there are any unit policies relating to women having to get out of the pool or not deliver in water in certain circumstances? (e.g. in the case of certain complications)
- Do you think that unit policies, procedures and guidelines support and encourage waterbirth? Why/why not? Why do you think that is?
- Do you know if there are any unit policies relating to monitoring of women in labour?
- Is continuous monitoring required in all/certain cases?
- Can women who need monitoring use a pool?

**Equipment and resources**

- Do you think that there are enough pools on the unit?
- Can women who want to use a pool always do so?
- Do you think that there are enough staff on the unit?
- Do you think unit staffing has an impact on pool use? How?
- Do you feel you can promote waterbirth to women, or would you be concerned that you might encourage this when there might not be a pool available?
- What do you advise women about pool availability?
- How well-known do you think pool availability is amongst local women?
- Do you think this plays a part in the number of women requesting a waterbirth because they know pools are / are not available?
- Do you know if there are instances of women having a home birth and hiring a pool just to guarantee being able to use a pool?
- Do you know if there are instances of women who would like to do this but can’t afford to?
- Do you know if there is an NHS (or private) pool rental service for home waterbirths?
- How is this advertised?
- How popular is it?
- Are there any issues with pool availability?
- What is the cost?

__________________________________________________________________________________

**End of interview**

- We’ve covered all of my questions – is there anything that we haven’t mentioned that you would like to say?
- Thank you for taking the time to talk to me today.
- Stop audio-recording.
