# Supplementary material for: Factors influencing water immersion during labour: qualitative case studies of six maternity units in the United Kingdom
Source: BMC Pregnancy Childbirth. 2020 Nov 23;20:719. doi: 10.1186/s12884-020-03416-7 (PMC7682119; doi:10.1186/s12884-020-03416-7)
Supplement: Supplementary file 5 — Additional file 5 Interview Topic Guide – Midwifery Support Workers. [file 12884_2020_3416_MOESM5_ESM.docx]

**Interview Topic Guide – Midwifery Support Workers**

**Introduction**

- Thank participant for agreeing to take part
- Are you happy for our conversation to be recorded for transcription and analysis?
- Start audio-recording
- The aim of this discussion is to explore your experiences and opinions in relation to the use of birth pools generally, and particularly to focus on the use of birth pools in this unit.
- What we talk about today will be kept confidential – only members of the research team will have access to the recording, and it won’t be labelled with your name. We might use some quotes from discussions in publications or presentations, but no names will be used.
- The aim of the discussion is to find out about your views and experiences, so there are no right or wrong answers.
- If there are any questions you don’t want to answer or if you would like to stop the recording or leave at any time, please let me know.
- Would you like to ask any questions before we start?

__________________________________________________________________________________

**Views of pool use for labour and birth**

- What is your view of women using a pool for labour and birth? / Do you think it is a good or a bad thing?
- Do you like supporting women who are having a waterbirth or labouring in water?
- Do some women prefer to get out of the pool to deliver?
- Can you see any benefits of waterbirth or using a pool during labour?
- Do you think there are any negative aspects or risks?
- Do you think there should be more or fewer waterbirths on the unit? Why?
- How does pool use affect your day-to-day work?
- Are waterbirths harder work or more difficult for you or for midwives than births on dry land?
- In terms of monitoring / delivery / physically?
- How do you think waterbirth and using a pool during labour is viewed by staff on the unit?
- By midwives?
- By midwifery managers?
- By consultants?
- Is waterbirth ever discussed on the unit?
- Do the risks or benefits of waterbirth tend to dominate discussions?
- Do you think some staff see waterbirth as being an added risk with no benefits?
- Do you think using a pool during labour is seen as being part of routine care or as being unusual?
- Has the way waterbirth is viewed on the unit changed over the last few years?
- Do all staff view waterbirth in the same way, or do you think different members of the team have different views of waterbirth?
- Are there any waterbirth ‘champions’ on the team?
- Is there anyone particularly against waterbirth?
- Who makes the decisions as to whether a woman uses a pool or gives birth in water?
- When women can get in the pool? / If/when they have to get out?
- Generally, do you feel that women can have a waterbirth on the unit if they wish to?
- Do you think that women who give birth at home are more likely to use a pool? Why?
- Have you noticed any changes over time to staff attitudes to waterbirth
- Do you think women are more likely to ask to use a pool now than X years ago?

**Women’s awareness of pool use as an option for labour and birth**

- Do you think women are aware of the option to use a pool before they arrive at the unit in labour?
- Do you know how they find out about it?
- When women have been admitted to the unit in labour, do you think they are aware of the option to use a pool?
- How visible are the pools on the unit? Is there one in every delivery room?
- Do you know what information women are given in pregnancy about the unit?
- Do they have a leaflet about the unit? Does it include pictures/information about pools?
- Do they have a tour of the unit? Would this include seeing a birth pool?
- What do women generally know about waterbirth?
- Are there any particular concerns or questions they raise about waterbirth?
- Do they have any misconceptions about waterbirth?
- Do midwives generally encourage women to try using a pool for labour and/or birth, or do they tend to offer pools only to women who request one?
- How proactive do you think women have to be to have a waterbirth on the unit?
- Roughly what proportion of women do you think request to use a pool? Why do you think this is?
- If women request to use a pool, are they usually able to use one?
- Are there certain groups of women who you think are more or less likely to request to use a pool?
- Are there certain groups of women who you think are more or less likely to get access to a pool?

**Criteria for pool use and how these are applied**

- Do you know if there are any unit policies, procedures or guidelines to follow relating to pool use?
- Do you know whether there are any groups of women who are not allowed to use a pool on the unit? Why?
- Do you know if there any groups of women who are only allowed to use a pool under certain conditions (e.g. only if they have monitoring or if they leave the pool to give birth)? Why?
- Can women who need monitoring use a pool?
- Do you know if there are there any unit guidelines relating to when women can get into the pool? (e.g. when x cm dilated)
- Do you know if there are any unit guidelines relating to women having to get out of the pool or not deliver in water in certain circumstances? (e.g. in the case of certain complications)
- Do you think there are any ‘unwritten’ policies, procedures or guidelines relating to pool use on the unit?
- Do you think what happens in practice on the unit tend to stick to the guidelines, or do staff sometimes tweak them?
- Can any of the guidelines be overruled? (e.g. by women’s choice / in certain cases / by certain staff)
- How helpful do you think the unit policies, procedures or guidelines are?
- Are there any problems with them? (e.g. are they too restrictive/inflexible?)
- Do you think that unit policies, procedures and guidelines support and encourage waterbirth? Why/why not? Why do you think that is?
- (If there have been any rises/falls in waterbirth rates on the unit) – Why do you think this is?

**Equipment and resources**

- How many pools do you have on the unit?
- How many are in working order and can be used?
- (If applicable) – What is the problem with the pool/s that can’t be used?
- Are there enough pools? Can women who want to use one always do so?
- (If few pools) – Do you know why this is?
- How long does it take to get a pool ready for the next patient after it has been used?
- What is the process?
- Who does this?
- Are there any issues with this?
- How long does it take to fill the pool?
- Has the length of time the pool takes to fill prevented some women from using it?
- At what point is the pool filled? (e.g. when the woman phones in, when they are x cm dilated, etc.)
- Are there any technical issues with using the pool? (e.g. temperature issues, etc.)
- Are there any physical issues with supporting women who are using the pool? (e.g. bad backs, etc.)
- Are there enough staff on the unit?
- Do you think staffing impacts on pool use?
- Are there several different types of pool on the unit? (e.g. some inflatable)
- (If yes) – Which type do you prefer / not like? Why?
- Are there any technical issues with any of them?
- Does the unit have waterproof monitoring equipment?
- Is this readily available? (If no) – Why?
- Are there any technical issues with it?
- Does it work in all rooms with a pool?
- What are the rooms with pools like?
- How do they compare to the rooms without pools?
- If pool rooms are ‘nicer’, are they sometimes used for women who don’t use the pool?
- Where are the pools located?
- Does the location of the pools cause any issues?
- Do you think pools are used efficiently on the unit?
- Are there often pools that aren’t being used? Why do you think this is?
- Are there sometimes women in rooms with pools waiting for discharge, etc.?
- Could pool use be increased with better management of resources?
- Are there any other issues relating to equipment and resources for pool use?

__________________________________________________________________________________

**End of interview**

- We’ve covered all of my questions – is there anything that we haven’t mentioned that you would like to say about the use of birth pools?
- Thank you for taking the time to talk to me today.
- Stop audio-recording.
