# Supplementary material for: Factors influencing water immersion during labour: qualitative case studies of six maternity units in the United Kingdom
Source: BMC Pregnancy Childbirth. 2020 Nov 23;20:719. doi: 10.1186/s12884-020-03416-7 (PMC7682119; doi:10.1186/s12884-020-03416-7)
Supplement: Supplementary file 6 — Additional file 6 Interview Topic Guide – Obstetricians and Neonatologists. [file 12884_2020_3416_MOESM6_ESM.docx]

**Interview Topic Guide – Obstetricians and Neonatologists**

**Introduction**

- Thank participant for agreeing to take part
- Are you happy for our conversation to be recorded for transcription and analysis?
- Start audio-recording
- The aim of this discussion is to explore your experiences and opinions in relation to the use of birth pools generally, and particularly to focus on the use of birth pools in this.
- What we talk about today will be kept confidential – only members of the research team will have access to the recording and it won’t be labelled with your name. We might use some quotes from discussions in publications or presentations, but no names will be used.
- The aim of the discussion is to find out about your views and experiences, so there are no right or wrong answers.
- If there are any questions you don’t want to answer or if you would like to stop the recording or leave at any time, please let me know.
- Would you like to ask any questions before we start?

__________________________________________________________________________________

**Views of pool use for labour and birth**

- What is your view of women using a pool for labour and birth?
- Overall, do you think waterbirths are a good or a bad thing? Why?
- Would you prefer it if women got out of the pool to deliver? Why?
- Can you see any benefits of waterbirth or using a pool during labour?
- Do you think there are any negative aspects or risks?
- In your opinion, are there any groups of women who shouldn’t have a waterbirth?
- How safe do you think waterbirths are overall?
- Do you think there should be more or fewer waterbirths on the unit? Why?
- How does waterbirth affect your day-to-day work?
- How do you think waterbirth and women using a pool during labour is viewed by your consultant colleagues?
- By midwives?
- Is waterbirth ever discussed amongst your colleagues?
- Do the risks or benefits of waterbirth tend to dominate discussions?
- Do you think some regard waterbirth as an added risk with no value?
- Do all staff view waterbirth in the same way, or do you think different members of the team have different views of waterbirth?
- Do you know if there are any waterbirth ‘champions’ on the team?
- Is there anyone particularly against waterbirth?
- Do you think the way waterbirth is viewed on the unit has changed over the last few years?
- Do you know whether waterbirths are seen as being part of routine care on the unit or as being unusual?
- Do you think that waterbirth gives staff less control?
- Do you think the unit is well-prepared to handle emergencies in the pool?

**Views of non-medicalised birth**

- Do you feel that birth is over- or under-medicalised on the unit? (e.g. that there is too much/ too little monitoring or intervention)
- Are natural births seen as the norm on the unit?
- Do you think there are any particular benefits of natural birth?

**Criteria for pool use and how these are applied**

- Do you know if there are any groups of women who are not allowed to use a pool? Why?
- Do you know if there are any groups of women who are only allowed to use a pool under certain conditions? (e.g. monitoring/leaving pool prior to giving birth)
- Do you know if high risk women can use a pool? How is ‘high risk’ defined?
- Are there any unit guidelines relating to women having to get out of the pool or not deliver in water in certain circumstances? (e.g. in the case of certain complications)
- What are the criteria for transfer to consultant-led care?
- Do you know what the policies, procedures or guidelines are relating to monitoring on the unit?
- Is continuous monitoring required in all/certain cases?
- Can women who need monitoring use a pool?

__________________________________________________________________________________

**End of interview**

- We’ve covered all of my questions – is there anything that we haven’t mentioned that you would like to say about the use of birth pools?
- Thank you for taking the time to talk to me today.
- Stop audio-recording.
