# Supplementary material for: Factors influencing water immersion during labour: qualitative case studies of six maternity units in the United Kingdom
Source: BMC Pregnancy Childbirth. 2020 Nov 23;20:719. doi: 10.1186/s12884-020-03416-7 (PMC7682119; doi:10.1186/s12884-020-03416-7)
Supplement: Supplementary file 7 — Additional file 7 Interview Topic Guide – Postnatal Women. [file 12884_2020_3416_MOESM7_ESM.docx]

**Interview Topic Guide – Postnatal Women**

**Introduction**

- Thank participant for agreeing to take part
- Are you happy for our conversation to be recorded for transcription and analysis?
- Start audio-recording
- The aim of this discussion is to explore your experiences and opinions in relation to the use of birth pools generally, and particularly to focus on the use of birth pools in this unit / your local maternity services.
- What we talk about today will be kept confidential – only members of the research team will have access to the recording, and it won’t be labelled with your name. We might use some quotes from discussions in publications or presentations, but no names will be used.
- The aim of the discussion is to find out about your views and experiences, so there are no right or wrong answers.
- If there are any questions you don’t want to answer or if you would like to stop the recording or leave at any time, please let me know.
- Would you like to ask any questions before we start?

__________________________________________________________________________________

I’d like to take down some brief information about you if that’s ok – this will be kept confidential.

- How long ago did you give birth?
- Where did you give birth? (Midwifery-led unit, consultant-led unit, at home)
- Did you use a pool or a bath during labour? If yes, did you give birth in water?

__________________________________________________________________________________

- When you were pregnant, did you discuss birth options with a midwife?
- Was waterbirth mentioned or discussed? Did you ask any questions about it?
- If yes – Did you also find out anything about waterbirth in other ways? (e.g. own research, antenatal classes, leaflets)
- If no – Were you aware that waterbirth was an option? How did you find out about it? (e.g. own research, antenatal classes, leaflets)
- Have any of your family or friends talked to you about their experiences of using a pool during labour? What did they say about it? Has anyone you know recommended using a pool, or not using a pool for labour and birth?
- (If not already covered) Did you attend any antenatal classes? Were these NHS classes or private classes (or both)?
- If yes – Was using a pool during labour mentioned in these classes (private/NHS or both)? What information were you given? Did you ask any questions about waterbirth?
- When you were pregnant, were you offered a tour of the unit?
- If yes - Did you see a birth pool on the tour? / Were you told there were birth pools?
- Were you given any other information about the unit (e.g. leaflets, from midwife)? Did this mention birth pools?
- Were you given any information about the likely availability of birth pools on the unit?
- Did you/would you have any concerns about using a pool during labour or giving birth in water?
- If yes – Did you talk to anyone about these concerns? What did they say?
- When you were in labour on the unit, were you made aware that you could use a pool?
- How? (e.g. told by midwife, poster, pool in room)
- Were you encouraged to use a pool?

**If used a pool:**

- Did you plan to use a pool in labour? Why?
- If yes, were you confident you would be able to access a pool?
- If no, how did you end up using the pool? (e.g. pool in room, midwife suggestion)
- How proactive did you have to be to be able to use the pool?
- Did you feel supported to use the pool?
- Were you able to get into the pool as soon as you wanted to?
- Were you asked to get out of the pool at any point? Why?
- Did you plan to give birth in the pool, or to get out for delivery?
- What actually happened?
- (If got out for delivery) – Why? Was this your choice?

**If had a home birth:**

- Why did you decide to have a home birth?
- Did wanting to use a pool play a part in your decision to give birth at home? Why?
- Did you rent or buy a pool?
- Where from?
- How did you find out about the pool hire service/shop?
- Did you shop around?
- Did you contact any pool hire services that had no pools available to hire?
- Can you remember what the pool/pool hire cost?
- (If applicable) – Are you aware of any NHS pool rental services? If yes, how did you find out about this?

**If didn’t use a pool:**

- Did you consider using a pool? Why/why not?
- If no - Do you think you could have used a pool if you had wanted to?
- If yes – What stopped you using a pool? (If applicable): Did you consider having a home birth to guarantee you would be able to use a pool?

Ask all:

- Do you think that there enough pools available at the unit?
- As far as you know, can women who want to use a pool always do so?

__________________________________________________________________________________

**End of interview**

- We’ve covered all of my questions – is there anything that we haven’t mentioned that you would like to say?
- Thank you for taking the time to talk to me today.
- Stop audio-recording.
