# Supplementary material for: Factors influencing water immersion during labour: qualitative case studies of six maternity units in the United Kingdom
Source: BMC Pregnancy Childbirth. 2020 Nov 23;20:719. doi: 10.1186/s12884-020-03416-7 (PMC7682119; doi:10.1186/s12884-020-03416-7)
Supplement: Supplementary file 8 — Additional file 8 Interview Topic Guide – Student Midwives. [file 12884_2020_3416_MOESM8_ESM.docx]

**Interview Topic Guide – Student Midwives**

**Introduction**

- Thank participant for agreeing to take part
- Are you happy for our conversation to be recorded for transcription and analysis?
- Start audio-recording
- The aim of this discussion is to explore your experiences and opinions in relation to the use of birth pools generally, and particularly to focus on the use of birth pools in this unit.
- What we talk about today will be kept confidential – only members of the research team will have access to the recording, and it won’t be labelled with your name. We might use some quotes from discussions in publications or presentations, but no names will be used.
- The aim of the discussion is to find out about your views and experiences, so there are no right or wrong answers.
- If there are any questions you don’t want to answer or if you would like to stop the recording or leave at any time, please let me know.
- Would you like to ask any questions before we start?

__________________________________________________________________________________

**Views of pool use for labour and birth**

- What is your view of women using a pool for labour and birth? / Do you think it is a good or a bad thing?
- Do you have any experience of supporting women who are having a waterbirth or labouring in water? How did you find this experience?
- Can you see any benefits of waterbirth or using a pool during labour?
- Do you think there are any negative aspects or risks?
- Do you think some women prefer to get out of the pool to deliver?
- Who is most likely to suggest or ask about using the pool when the woman comes into the unit? The midwife or the woman themselves?
- Do you think there should be more or fewer waterbirths on the unit? Why?
- How do you think pool use affects the day-to-day work of staff on the unit?
- Do you think waterbirths are harder work or more difficult for midwives than births on dry land?
- In terms of monitoring / delivery / physically?
- How do you think waterbirth and using a pool during labour is viewed by staff on the unit?
- By midwives?
- By midwifery managers?
- By consultants?
- Is waterbirth ever discussed on the unit?
- Do the risks or benefits of waterbirth tend to dominate discussions?
- Do you think some staff regard waterbirth as an added risk with no value?
- Do all staff view waterbirth in the same way, or do you think different members of the team have different views of waterbirth?
- Are there any waterbirth ‘champions’ on the team?
- Is there anyone particularly against waterbirth?
- How do these differences of opinion get resolved typically?
- Do you think using a pool during labour is seen as being part of routine care on the unit or as being unusual?
- Do you know who makes the decision as to whether a woman uses a pool or gives birth in water?
- When women can get in the pool? / If/when they have to get out?
- Do you think there is support from consultants for pool use?
- Do you think they can see any benefits of pool use?
- Do you think they prefer women to get out of the pool to deliver?
- Do you think they have any concerns about the safety of waterbirth or use of a pool in labour?
- Generally, do you feel that women can have a waterbirth on the unit if they wish?
- Is there anything that you think stops midwives offering waterbirth as a choice?
- How does staff support for pool use on this unit compare to support for pool use on other units you have worked on?
- Do you think that women giving birth at home are more likely to use a pool? Why?
- Do you feel that birth is over- or under-medicalised on the unit? (e.g. that there is too much/ too little monitoring or intervention)
- Do you think natural births are seen as the norm on the unit?

**Staff confidence, knowledge and experience**

- How knowledgeable do you feel about waterbirth?
- Have you been taught about waterbirth through your degree course or on placement?
- What have you been taught about it?
- Did you find this useful?
- Has waterbirth been seen as an important part of your training?
- Is there anything you feel you would like to know about waterbirth that you haven’t been taught?

**Women’s awareness of pool use as an option for labour and birth**

- Do you think women are aware of the option to use a pool before they arrive at the unit in labour?
- Do you think women tend to be aware of the option to use a pool once they have been admitted to the unit in labour?
- How visible are the pools on the unit? Is there one in every delivery room?
- What do women generally know about waterbirth?
- Do you know if there are any particular concerns or questions they raise about waterbirth?
- Do you know if they have any misconceptions about waterbirth?
- Do midwives on the unit generally encourage women to try using a pool for labour and/or birth, or do they tend to offer pools only to women who request one?
- How proactive do you think women have to be to have a waterbirth on the unit?
- If women request to use a pool, are they usually able to use one?
- Are there certain groups of women who you think are more or less likely to request to use a pool?
- Are there certain groups of women who you think are more or less likely to get access to a pool?
- Are women who give birth in this unit more or less likely to request using a pool than women on other units you have worked on? Why do you think this is?

**Criteria for pool use and how these are applied**

- Do you know if there are any unit policies, procedures or guidelines to follow relating to pool use?
- Do you know whether there are any groups of women who are not allowed to use a pool on the unit? Why?
- Do you know if there any groups of women who are only allowed to use a pool under certain conditions (e.g. only if they have monitoring or if they leave the pool to give birth)? Why?
- Can women who need monitoring use a pool?
- Do you know if there are there any unit guidelines relating to when women can get into the pool? (e.g. when x cm dilated)
- Do you know if there are any unit guidelines relating to women having to get out of the pool or not deliver in water in certain circumstances? (e.g. in the case of certain complications)
- Do you think there are any ‘unwritten’ policies, procedures or guidelines relating to pool use on the unit?
- Do you think what happens in practice on the unit tend to stick to the guidelines, or do staff sometimes tweak them?
- Can any of the guidelines be overruled? (e.g. by women’s choice / in certain cases / by certain staff)
- How helpful do you think the unit policies, procedures or guidelines are?
- Are there any problems with them? (e.g. are they too restrictive/inflexible?)
- Do you think that unit policies, procedures and guidelines support and encourage waterbirth? Why/why not? Why do you think that is?

**Equipment and resources**

- How many pools are there on the unit?
- Do you think there are there enough pools? Do you know if women who want to use one can always do so?
- Do you know how long does it take to get a pool ready for the next patient after it has been used?
- Do you know what the process is?
- Who does this?
- Are there any issues with this?
- Do you know how long it takes to fill the pool?
- Has the length of time the pool takes to fill prevented some women from using it?
- At what point is the pool filled? (e.g. when the woman phones in, when they are x cm dilated, etc.)
- Are there any regular or common technical issues with using the pool? (e.g. temperature issues, etc.)
- Are there any physical issues with supporting women who are using the pool? (e.g. bad backs, etc.)
- Are there enough staff on the unit?
- Do you think staffing impacts on pool use?
- Do you think there are there enough staff trained and experienced in waterbirth?
- What are the rooms with pools like?
- How do they compare to the rooms without pools?
- How do they compare to pool rooms on other units you have worked on?
- If pool rooms are ‘nicer’, are they sometimes used for women who don’t use the pool?
- Where are the pools located?
- Does the location of the pools cause any issues?
- Do you think pools are used efficiently on the unit?
- Are there often pools that aren’t being used? Why do you think this is?
- Are there sometimes women in rooms with pools waiting for discharge, etc.?
- Do you think pool use could be increased with better management of resources?

__________________________________________________________________________________

**End of interview**

- We’ve covered all of my questions – is there anything that we haven’t mentioned that you would like to say about the use of birth pools?
- Thank you for taking the time to talk to me today.
- Stop audio-recording.
